# Data mining analysis of human gut microbiota links Fusobacterium spp. with colorectal cancer onset

**DOI:** 10.6026/97320630015372

**Published:** 2019-05-30

**Authors:** Najla Kharrat, Mourad Assidi, Muhammad Abu-Elmagd, Peter N Pushparaj, Areej Alkhaldy, Leila Arfaoui, Muhammad Imran Naseer, Abdelfatteh El Omri, Safia Messaoudi, Abdelbaset Buhmeida, Ahmed Rebai

**Affiliations:** 1 1Laboratory of Molecular and Cellular Screening Processes, Centre of Biotechnologyof Sfax, Bioinformatics Group, P.O. Box: 1177,Sfax,3018 Tunisia; 2 Center of Excellence in Genomic Medicine Research, King Abdulaziz University, Jeddah, Saudi Arabia; 3 Medical Technology Department, Faculty of Applied Medical Sciences, King Abdulaziz University, Jeddah, Saudi Arabia; 4 4School of Biological Sciences,University of East Anglia, Norwich, NR4 7TJ, UK; 5 Clinical Nutrition Department, Faculty of Applied Medical Sciences, King Abdulaziz University, Jeddah, Saudi Arabia; 6 Department of Biological Sciences, Faculty of Science, King Abdulaziz University, Jeddah, Saudi Arabia; 7 Forensic Biology Department, College of Forensic Sciences, Naif Arab University for Security Sciences, Riyadh, Saudi Arabia

**Keywords:** Colorectal cancer, microbiome, bacteria, Fusobacterium spp, IDA method, biomarker

## Abstract

Gut microbiota and their metabolites play a vital role in colon health and disease. Accumulating evidence suggests that the gut microbiota contributes to the risk of colorectal cancer (CRC). However, the role of a specific microbial community together with their metabolites contributing to the risk, initiation and progression of CRC is still unknown. Hence, we used a Bayesian Networks in combination with the IDA (Intervention calculus when the DAG is absent) to generate a graphical model that allows causal relationships to be inferred from observational data. Results from the analysis of publically available datasets showed that four species: Fusobacteium, Citrobacter, Microbacterium and Slaxkia have estimated non-null lower bounds of causal effects of CRC. These findings support the hypothesis that specific bacterial species (microbial markers) act in concert with locally modified microbiota to cause or influence CRC progression. Additional comprehensive studies are required to validate the potential use of F. nucleatum, Citrobacter as well as Slackia as microbial biomarkers in CRC for prevention, diagnosis, prognosis and/or therapeutics.

## Background

In an era marked by the emergence of complex diseases as cancer, diabetes, obesity and cardio-vascular diseases with heavy economic and social burden, better understanding of the diseases complexity, causes and progression are essential to tailor more effective approaches for both prevention and therapy 1. The innovation of high-throughput technologies has allowed better understanding of the interactions of each individual genomic makeup with both his/her environment and lifestyle to foster health or initiate diseases. These systems medicine approaches using mainly advanced metagenomics and bioinformatics tools have revealed the importance of microbiome - as a living component of the environment in both health and disease 2. Human microbiota is reported to be implicated in ~20% of human malignancies 3. Accumulating evidences support the relationship between infective agents and human cancer suggesting the important role of bacterial species in the pathogenesis of cancer 4-6. Studies have reported an association between certain bacterial species and different types of cancers 7, 8.

The human gut is hosting a large, diverse and dynamic microbiota species shaped according to each individual genomic background, environment and lifestyle mainly diet and exercise. The imbalance of gut microbiota hemostasis (dysbiosis) is reported to induce several diseases 9. Recent works have provided mechanistic evidence for the involvement of gut microbiota in the onset of CRC. 10-12. The mechanisms through which microbes initiate carcinogenesis can be assessed by different parameters including DNA damage, inflammation, cell proliferation and migration, activation of pro-carcinogenic pathways or production of genotoxins 13, 14. Many bacterial species have been shown to be involved in these mechanisms. Among these species is S. gallolyticus subspecies, which colonizes and invades colon tumors leading to enhanced tumor growth through inflammatory signalling 8. Another species is Enterotoxigenic bacteroides fragilis which alters colonic epithelial cells structure and functions by enhancing Wnt/β catenin signaling leading to increased colonic carcinoma cell proliferation and expression of the proto-oncogene MYC 15. The bacterial strain Escherichia coli, which contains polyketide synthase (pks) island, induces double strand DNA breaks through the production of genotoxin colibactin, leading to promoting colon carcinogenesis 16. Moreover, Fusobacterium spp. induces an expansion of myeloid-derived immune cells in the tumor microenvironment and upregulates inflammatory genes in colon tumors 17.

It is still not clear, whether a specific species, a microbial community, or both could initiate the above-mentioned biological events and promote CRC. Moreover, dietary metabolites derived from gut microbiota were reported to affect colon health and play a significant role in the aetiology of CRC. Some studies indicated that the microbial metabolites such as phenol, ammonia, primary and secondary bile acids could act as pro-carcinogenic in the colon, whereas other metabolites found to have a positive effect by suppressing the inflammation, inhibiting proliferation, as well as modulating the differentiation and gene expression in the colonic epithelium cell. These metabolites include the short chain fatty acids (SCFA) such as butyrate, acetate, and propionate and polyphenolic compounds. It is possible that hormonal secretion or the production of bacteriocins acts on the balance of this community, depending on the host physiology as a response to a host's diet or ingested pharmaceuticals. Despite this fact, so far no signature of distinct bacterial colonization patterns in CRC patients was established. The standard methods to discover these patterns are based on the statistical analysis of the diversity and community structure between individuals (healthy vs patients) and reflect enrichment in tumors biopsies versus healthy tissues. However, these results are merely based on classical statistical analyses such as pairwise relationship (t-test) or regression techniques and these are neither sufficient to model the complexity of these mechanisms nor enough to establish a causal relationship between species prevalence and cancer. Most importantly, these methods are unable to determine the real causal species implicated in CRC among those that show different abundances in tumors and healthy tissues.

In this context, there is a real need for a model that integrates biological data of multiple sources to fit and infer causal relationships. In this pilot study, Bayesian Networks in combination with the 'Intervention-calculus when the DAG is Absent' (IDA) method (method reported by Maathuis et al. (2009) and Maathuis et al. (2010) 18, 19) was used in order to generate a graphical model that allows causal relationships to be inferred from observational data. This approach has been already used in other contexts, for example, to discover the miRNA-mRNA causal regulatory relationships from observational data 20. Here, the IDA method was applied on two independent studies suitable for causal inference relationships: Marchesi et al. (2011) 13 and Zeller et al. in 2014 21. The goal was to identify key species that are likely to be causal agents of CRC.

## Methodology

The structure of conditional independence relationship between random variables were often presented as DAG (Directed Acyclic Graph) which vertices represent random variables encode conditional dependence of the enclosing vertices. This structure is applied by do-calculus but it is not always the case. Maathuis et al. (2009, 2010) 19, 18 estimated causal effect when the DAG is absent (IDA). This IDA method (Intervention calculus when the DAG is Absent), included two main phases: (i) to learn a causal structure from observational data and (ii) to apply the do-calculus to infer causal effects. In this study, bacteria species are nodes or variables in the model, and observational data are the abundance profiles of the species in malignant and adjacent non-malignant tissues in the study of Marchesi et al. (2011) 13 and in two population samples (healthy versus patients) in the case of Zeller et al. (2014) 21.

**Figure 1:**
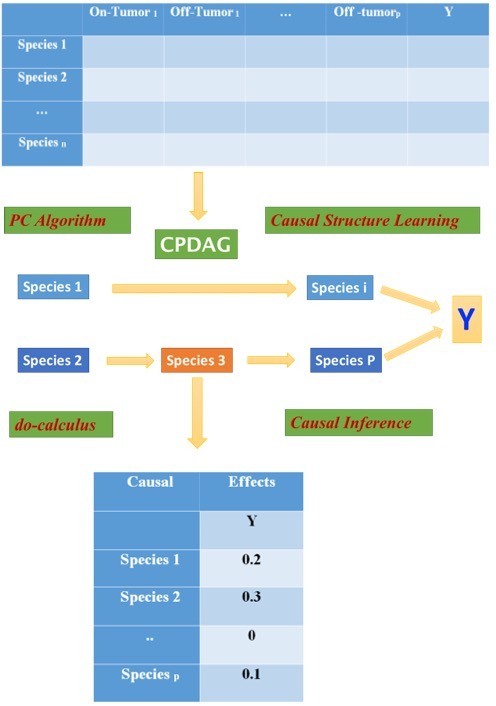
Schematic flowchart showing the main steps of the IDA Method

The input dataset has p+1 variables: p which are the abundances of p species are quantitative and one binary response variable Y (tumour/non-tumour or patient/healthy). In the first phase, the causal structure in the form of a CPDAG (Completed Partially Direct Acyclic Graph) is learnt from data, by applying the PC algorithm. We used partial correlation as a conditional independence test for the PC algorithm. However, when the number of nodes in the CPDAG was large, we applied the theorem cited by Le et al. (2013) 20 to reduce the search space of possible DAGs. In the second phase, the do-calculus is used to estimate causal effects of a given species 'i' on the target variable 'Y'. In our case, the causal effect of a species 'i' is estimated as the binary logistic regression coefficient of 'Y' on the species and its parents in the DAG. This coefficient is the lower bound of the causal effect of species 'i' on 'Y' 19. If a species had a lower bound >0, we concluded that it was a causal agent otherwise (if it was 0) we cannot exclude a causal effect (Figure 1).

### Algorithm:

Step1: First, we divided the data set as per categories (Normal vs Cancer) and identified the differential abundance of taxa across categories. We assumed that the taxa with little or zero change in abundance between categories paly a minimum role in the biological processes and thus omitted.

Let X1, . . . ,Xm represent taxa abundance and 'Y' the status category (binary variable : normal versus cancer) of taxa across categories. We have a dataset for the (m+1) variables. Step 2: Use the PC algorithm to estimate the CPDAG G of the (m+1) variables and the conditional dependencies of the variables. We used partial correlation as a conditional independence test for the PC algorithm, as the partial correlations were easy to implement in a high-dimensional dataset.

Step 3: Estimated the causal effects of each taxa on each category. Naturally, we can identify all possible DAGs in the CPDAG, and estimated the causal effects with each DAG. However, when the number of nodes in the CPDAG was large, we reduced the search space of possible DAGs.

Step 4: Output the taxa causal effects. For each taxa, the outcome of Step 3 was considered as an array of multisets, and each multiset contains all causal effects of the taxa on the status. With each of the multisets, in this step, we selected the causal effect value with the smallest absolute value, and outputted it as the causal effect of taxa on the status.

### Description of the datasets used:

Only two independent data, related to the colorectal cancer where the abundance specific taxa available, were used for applying the IDA method. In the first study by Marchesi et al. (2011) six patients underwent resections for primary colon adeno-carcinoma 13. The differential abundance of specific taxa between the microbiomes colonizing colon tumor tissue versus their adjacent non-malignant mucosa is shown.

In the study by Zeller et al. (2014), we used the 156 participants recruited in France, who underwent colonoscopy to either diagnose colorectal neoplasia in the form of adenoma(s) (polyps) or CRC, or confirmed for the absence of cancer. Carcinomas were further classified according to established staging systems (AJCC and TNM) 22. The microbial community metagenomics analysis was carried out on stool samples for 88 control and 53 CRC patients.

The significance of the difference between abundance of specific taxa was performed by t-test within R language. To reduce the number of variables, a binary logistic regression was applied to the significant associated species. The latter were used as an input file for the IDA method.

## Results

A network model representation of the relationship between CRC microbiota species:

### First study:

**Figure 2:**
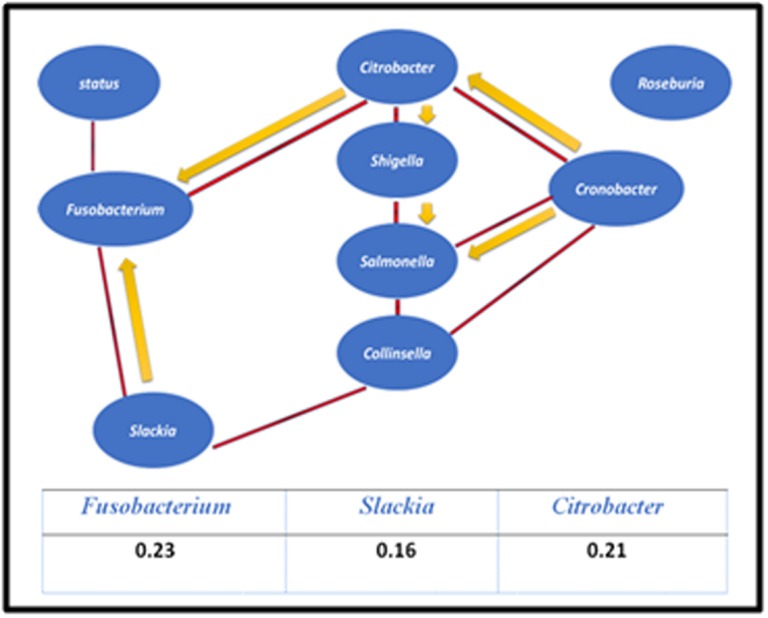
Network model for the study of Marchesi et al. (2011). Numbers in the table correspond to regression coefficients.

For the first study of Marchesi et al., the network structure obtained is illustrated in Figure 2. All estimated lower bounds of causal effects of genera on cancer status were 0 except for Fusobacterium (0.23); Citrobacter (0.21) and Slackia (0.16).

### Second study:

**Figure 3:**
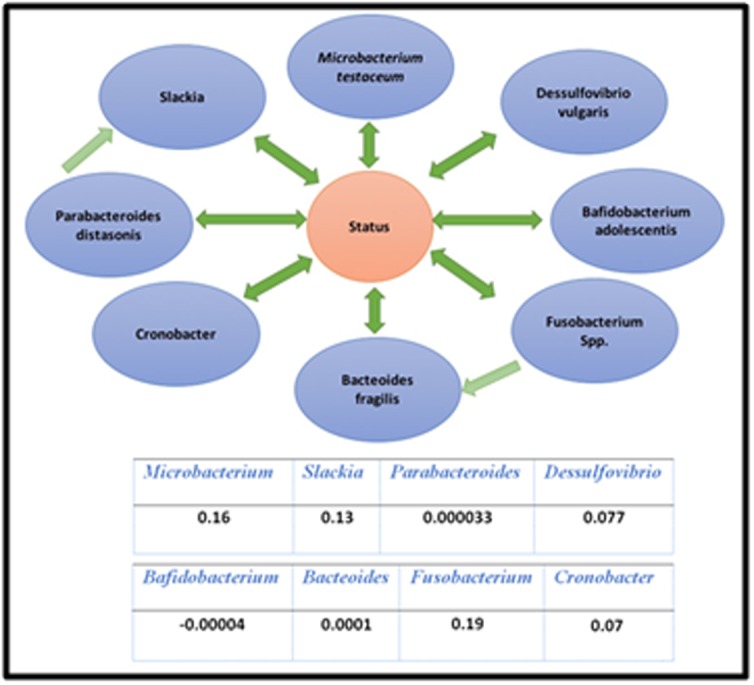
Network structure for the study of Zeller et al. 2014. Numbers in the table correspond to regression coefficients.

In the second study of Zeller et al., over 1500 species were reported. We selected only those that have significant association with cancer using t-test. Selecting only species showing significant associations with cancer by multivariate binary logistic regression refined the list. The selected shortlist of species was then used to apply IDA. The graph structure generated also enhanced the importance of Fusobacterium spp. as a causal species involved in the colorectal cancer with the highest coefficient regression followed by Microbacterium testaceum and Slackia (with 0.19, 0.16 and 0.13, respectively, Figure 3). All the other species like (Parabacteroides distasonis, Dessulfovibrio vulgaris, Bafidobacterium adolescentis, Bacteoides fragilis, Cronobacter) seemed to play a role in the carcinogenesis of CRC but with moderate effect.

For the two-independent analysis, the common hubs of the two networks were the two species Fusobacterium spp. and Slackia. In fact, these two species seemed to be involved together in biomolecular perturbations/causing CRC.

### Pathways enrichment:

Our results indicated that there was substantial evidence that the four species detected by the IDA method had causal effects in carcinogenesis pathways. Pathway information captures knowledge of biological processes at the molecular level and can be considered as an important tool for interpreting the growing amount of biological data with pathway enrichment analysis. Here, we used Biocyc software (https://biocyc.org) to search pathways across the four species 23. Since different strains were available for these species, only strains described in human colorectal cancer and available in biocyc.org were chosen: Fusobcterium nucleatum animalis 11_3_2; Microbacterium testaceum 87StLB037; Slackia exgua ATCC 700122 and Citrobacter Koseri ATCC BAA-895.

This refinement allowed dividing pathways into groups based on their biological functions, and based on the classes of metabolites that they produce and/or consume. Here, we reported the 7 classes of pathways generated by biocyc database for each taxa (Table 1). We counted only the shared pathways between organism pairs. Interestingly, the Fusobacterium was shown to be the main causal bacteria in the two graphs generated per IDA method for the two studies. Furthermore, we found that the pathways shared between Fusobacterium and the other organisms were classified in accordance with the regression coefficient calculated via IDA method. In fact, F. nucleatum animalis 11_3_2shared 145, 144 and 108 pathways within C. koseri ATCC BAA-895, M. testaceum StLB037 and S. exigua ATCC 700122 respectively (Table 2).

**Figure 4:**
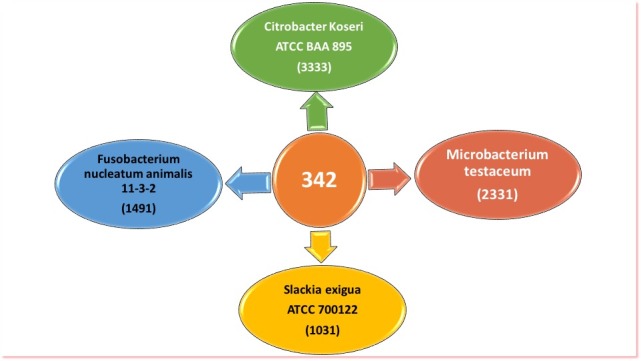
Individual and shared distribution of orthologue proteins in the four microbial species

So far, not all molecular interactions in a pathway have been associated with a corresponding known gene(s) that was completely identified in the human genome. This explains why pathway holes may exist. They may represent true enzymatic or metabolic functions in the organism for which the corresponding human gene has not yet been identified, or they could represent false positive pathway predictions, or particular cases in which the pathway in this organism differs slightly from the reference pathway in (MetaCyc). Table 3 counts all the pathway holes in each organism database, and classifies pathways based on their number of pathway holes. Fusobacerium process the high percentage of holes pathways. We try to explain this specific pathway prediction via data driven of functional homology. We found only 342 orthologous proteins shared within the four species (Figure 4).

## Discussion

Human microbiota is nowadays recognized as a key player in wellness and disease. Despite the huge progress in OMICs technologies and systems biology, the comprehensive study of microbial species types, abundance, diversity, interactions, and contribution in health and diseases is still challenging 24, 25. More powerful tools, integrative and multidisciplinary approaches are required to demystify such complexity in order to set up more predictive, preventive, participative, precise and cost-effective systems medicine that enhance the society wellness and prevent complex and chronic diseases 26. Increasing evidence suggests a possible role for certain bacteria in colorectal carcinogenesis 27, 28. However, the association of the composition of the microbiome with the host factors such as gender, age, smoking, diet, exercise, and oncogenes polymorphisms is still poorly understood 27, 28, 17, 11. In fact, the statistical relationships using most computational methods revealed associations or correlations but not causality, which is yet to be established. In this study, we used an alternative approach, namely IDA method 19, 18, which can reveal causal relationships between bacteria and CRC status. Results obtained from the IDA analysis of the two previously described studies were in agreement with all previous association studies conducted in CRC. In fact, metagenomic analysis showed a significant enrichment of Fusobacterium spp and particularly F. nucleatum. This bacterium enriched in CRC was significantly more abundant in adenoma CRC patients compared to healthy individuals. This strain of Fusobacterium was associated within specific human expression profile, which was not shared by other bacterial species present in high number in the colon 29. Furthermore, F. nucleatum appeared to be the dominant phylotype bacteria associated positively with lymph node metastases 30. The abundance of Fusobacterium has been previously correlated to the expression of myeloid associated genes as well as to NF-kB driven inflammatory genes in human CRC 31. It has been shown that Fusobacterium expanded myeloid-derived immune cells which inhibit-T-cell proliferation and induced T-cell apoptosis in murine models 31. Increased amounts of tissue F. nucleatum DNA have been reported to be associated with proximal tumor location, higher depth of tumor invasion, poor tumor differentiation, higher microsatellite instability in univariate and multivariate analysis, hypermethylation independent CpG island methylator phenotype (CIMP) and BRAF mutation status in univariate analysis 32-34. In the study carried out by Mima et al. (2015) 34, the amount of F. fusobacterium DNA in CRC tissue has been positively associated with CRC-specific mortality, independent of clinical, pathological and major tumor molecular features. This proportion gradually increased from the proximal to distal segments and potentiates colonic neoplasia development. F. nucleatum suppressive modulates the tumor-immune microenvironment by activating Wnt signaling pathway in CRC cells and may promote colorectal tumor growth and mucosal priming for neoplasia 28. In addition, F. nucleatum produced the adhesion molecule FadA that invaded the epithelial cells, and promoted pro-oncogenic response. Once produced, this virulent factor binds to E-cadherin mediating attachment at epithelial cells inducing invasion of F. nucelatum, which activates β catenin signalling. This pathway was also shared with other bacteria, approved by our model in the study of Marchesi et al. 13 at the second level, the Citrobacter. In another study, C. rodentium induced transmissible murine colonic hyperplasia in murine models associated with Wnt/βcatenin, Notch, PI3K and NFκB pathways 35. It appears that these two linked bacterial infections, Fuso bacterium and Citrobacteria, were involved in CRC. For the third specie, Slackia, which was reported by our model, has been shown to cause meningitis in patients with CRC in rare but serious complications. In fact, its presence is concomitant with other bacteria such as Saureus, E. coli, B. fragilis, C. ramosum, Prevotella/Porphyromonas spp., and Fnucleatum 36. However, further studies are required to confirm this suggested pathogenicity and clinical significance of Slackia in this case.

The second causal effect reported by Zeller et al. 21 where Microbacterium testaceum described as a causative agent of infections in immune compromised patients i.e. cancer patients. Its pathogenicity in this context remains unknown and therefore lacking enough clinical evidence to consider it as a clinical marker of CRC evolution as reported by our model. However, this bacterium could be considered as an opportunistic human pathogen in immune compromised patients 37 but further investigations are needed to support this hypothesis. The presence of orthologous proteins attested by the analysis of enrichment pathways shared between the four-species supports interactions acting on the same process, but a high percentage of holes pathways demonstrated the lack of evidence for valuable pathways incriminated in carcinogenesis of CRC 23. It is obvious that there was substantial evidence that the signalling cascade is involved in many other molecular phenomena. Further studies are required to demystify these still unknown functions and/or pathways.

Our model allowed the discussion of disease causation scenarios and supported the hypothesis in which limited bacterial species act in concert with locally modified gut microbiota to cause CRC. The concept of keystone species or a microbial driver that recruits a consortium of disease facilitating a microbial community to initiate the biologic events causing CRC could be envisioned. However, our model could be improved by adding confounding factors (i.e. gender, BMI, age, smoking, diet, exercise) to validate the potential use of F. nucleatum, Citrobacter and/or Slackia as early detection and/or diagnostic microbiota markers in CRC screening. Our results are in line with several recent evidence-based studies linking the causality, the onset and progression of CRC to severe imbalance to gut microbiota 14, 38-40.

Taken together, these findings lay foundation and provide insights for future studies to develop strategies for CRC prevention, screening (for diagnosis) and treatment through targeting the driver gut microbiota. A better understanding of gut microbial communities' diversity, interactions and roles is crucial to demystify the underlying causes of complex and chronic diseases and promote innovative proactive approaches to alleviate their burden on human health, society and economy. The interpretation of these studies also requires a better understanding of interindividual variations, heterogeneity of microbial communities along and across the GI tract, functional redundancy and the need to distinguish the cause from effect in states of dysbiosis 39.

## Conclusion

This pioneering study provided supportive evidence of crucial role of specific gut microbial species and their metabolites in CRC risk and onset. Further comprehensive studies using both high throughput metagenomic approaches and advanced computational tools are required to demystify the host-microbiota interactions and enhance our understanding of their impacts on gut integrity, microbiota hemostasis and human health.

## Conflict of Interest

The authors declare no conflict of interest.

## Ethics approval and consent to participate:

Not applicable

## Figures and Tables

**Table 1:** The root of a classification hierarchy for metabolic pathways in the four species

Pathway classes	Fusobcterium nucleatumanimalis	Microbacterium	Slackia exgua	Citrobacter koseri
	11_3_2	testaceum	ATCC 700122	ATCC BAA-895
		StLB037		
Activation/ Inactivation/inter conversion	2	2	1	3
Bio synthesis	161	209	136	220
Degradation/utilization/assimilation	59	121	47	156
Detoxification	4	7	2	3
Generation of precursor metabolites and energy	20	47	22	52
Metabolic Clusters	4	8	6	5
Super pathways	46	78	33	78
Total	194	303	173	352

**Table 2:** Overview of the shared pathways between the four species

Pathways shared by organism pairs	C. koseri	F. nucleatum animalis	M. testaceum	S. exigua
	ATCC BAA-895	11_3_2	StLB037	ATCC 700122
Citrobacter koseri ATCC BAA-895	331	145	213	128
Fusobacterium nucleatum animalis 11_3_2	145	188	144	108
Microbacterium testaceum StLB037	213	144	290	131
Slackia exigua ATCC 700122	128	108	131	167

**Table 3:** Summary of pathway holes for each species

Pathway Holes	C. koseri	F. nucleatum animalis	M. testaceum	S. exigua
	ATCC BAA-895	11_3_2	StLB037	ATCC 700122
Number of Pathway Holes	100	254	176	178
Pathway Holes as a percentage of total reactions in pathways	12%	44%	23%	35%
Pathways with No Holes	258	79	185	90
Pathways with 1 Hole	45	44	59	39
Pathways with 2 Holes	19	24	21	14
Pathways with 3 Holes	5	13	13	3
Pathways with 4 Holes	4	11	5	7
Pathways with 5 Holes	0	9	2	7
Pathways with > 5 Holes	0	8	5	7
Total Pathways with Holes	73	109	105	77
